# Tonsillar Fullness Mimicking Malignancy Secondary to a Cervical Osteophyte: A Case Report

**DOI:** 10.7759/cureus.108387

**Published:** 2026-05-06

**Authors:** Jonah Zeitlin, Olivia Matz, Gary Kwartowitz, Haley Wilenzick, Lauren Hecht

**Affiliations:** 1 Otolaryngology - Head and Neck Surgery, McLaren Oakland Hospital, Pontiac, USA; 2 Otolaryngology - Head and Neck Surgery, Binghamton University, Binghamton, USA

**Keywords:** cervical osteophyte, extrinsic compression, globus sensation, head and neck imaging, oropharyngeal mass, oropharynx, squamous cell carcinoma mimic, tonsillar asymmetry

## Abstract

Tonsillar asymmetry and firmness on examination often raise concern for underlying malignancy, particularly squamous cell carcinoma or lymphoma. However, rare benign etiologies may mimic these findings and lead to diagnostic uncertainty. We present the case of an 83-year-old female who presented with globus sensation and was found to have left-sided tonsillar fullness with firmness on physical exam, raising concern for malignancy. Flexible laryngoscopy was unremarkable. Computed tomography of the neck demonstrated a prominent anterior cervical osteophyte at the C1-C2 level, causing mass effect on the left palatine tonsil, resulting in anteromedial displacement and mild oropharyngeal narrowing. No mucosal lesions or lymphadenopathy were identified. This case highlights an unusual cause of tonsillar asymmetry and underscores the importance of imaging in distinguishing malignant from benign etiologies.

## Introduction

Tonsillar asymmetry in adults raises concern for malignancy, particularly in the presence of red flag symptoms such as rapid enlargement, suspicious mucosal changes, firm consistency, cervical lymphadenopathy, throat pain, adjacent tissue involvement, or systemic symptoms [1]. Data suggest that rapid tonsillar enlargement is the most predictive feature, with a specificity of 96.4% for malignancy. In contrast, when asymmetry is present in isolation without additional red flag features, the likelihood of malignancy is less than 1% [2]. The presence of associated lymphadenopathy increases the risk of malignancy to 38.5% [2].

In adults undergoing tonsillectomy for suspicious findings such as tonsillar asymmetry, tonsillar mass, or concern for malignancy, squamous cell carcinoma is the most commonly identified malignancy, occurring in 32.8% of cases [3]. The majority of these cases are human papillomavirus (HPV)-associated, with 95.5% demonstrating p16 positivity [3]. In series specifically examining asymmetric tonsils, lymphoma has been identified as the most common malignancy [4].

Despite concerns for malignancy when a patient presents with tonsillar asymmetry, non-malignant processes can occasionally mimic these findings. Cervical osteophytes are common degenerative changes of the spine but are rarely implicated in oropharyngeal mass effect. A study of 225 patients demonstrated a significant correlation between globus sensation and the total number of cervical osteophytes, particularly at the C4-5, C5-6, and C6-7 levels; however, these findings were attributed to posterior pharyngeal wall compression and did not present as tonsillar fullness [5]. We present a case in which a cervical osteophyte caused apparent tonsillar fullness, mimicking a neoplastic process.

## Case presentation

An 83-year-old female with a past medical history of hyperlipidemia presented to her primary care physician with mild globus sensation for several weeks and the subjective perception of a mass in the posterior oropharynx.

**Figure 1:**
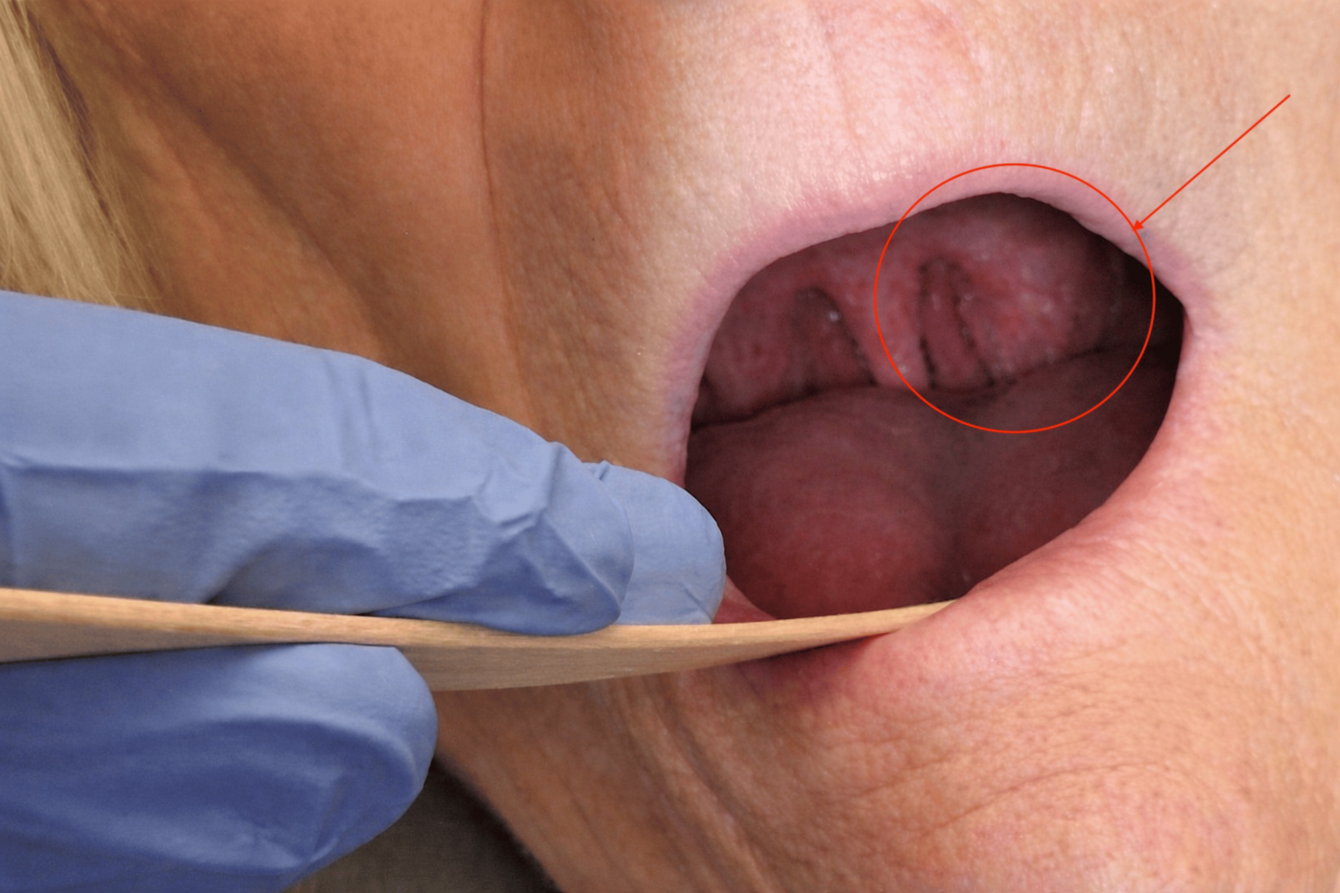
Oropharynx exam demonstrating left tonsillar fullness.

She was referred to otolaryngology for further evaluation. On examination, there was notable fullness of the left palatine tonsil as shown in Figure 1. Palpation revealed a firm, non-tender area without additional regions of induration. These findings raised concern for possible malignancy. She denied a history of tobacco use, alcohol use, or known HPV exposure.

Flexible fiberoptic laryngoscopy demonstrated a widely patent airway, normal bilateral true vocal fold mobility, and no visible mucosal lesions or masses within the oropharynx, hypopharynx, or larynx. There was no palpable cervical lymphadenopathy. The patient denied systemic symptoms, including weight loss, fevers, or night sweats.

**Figure 2:**
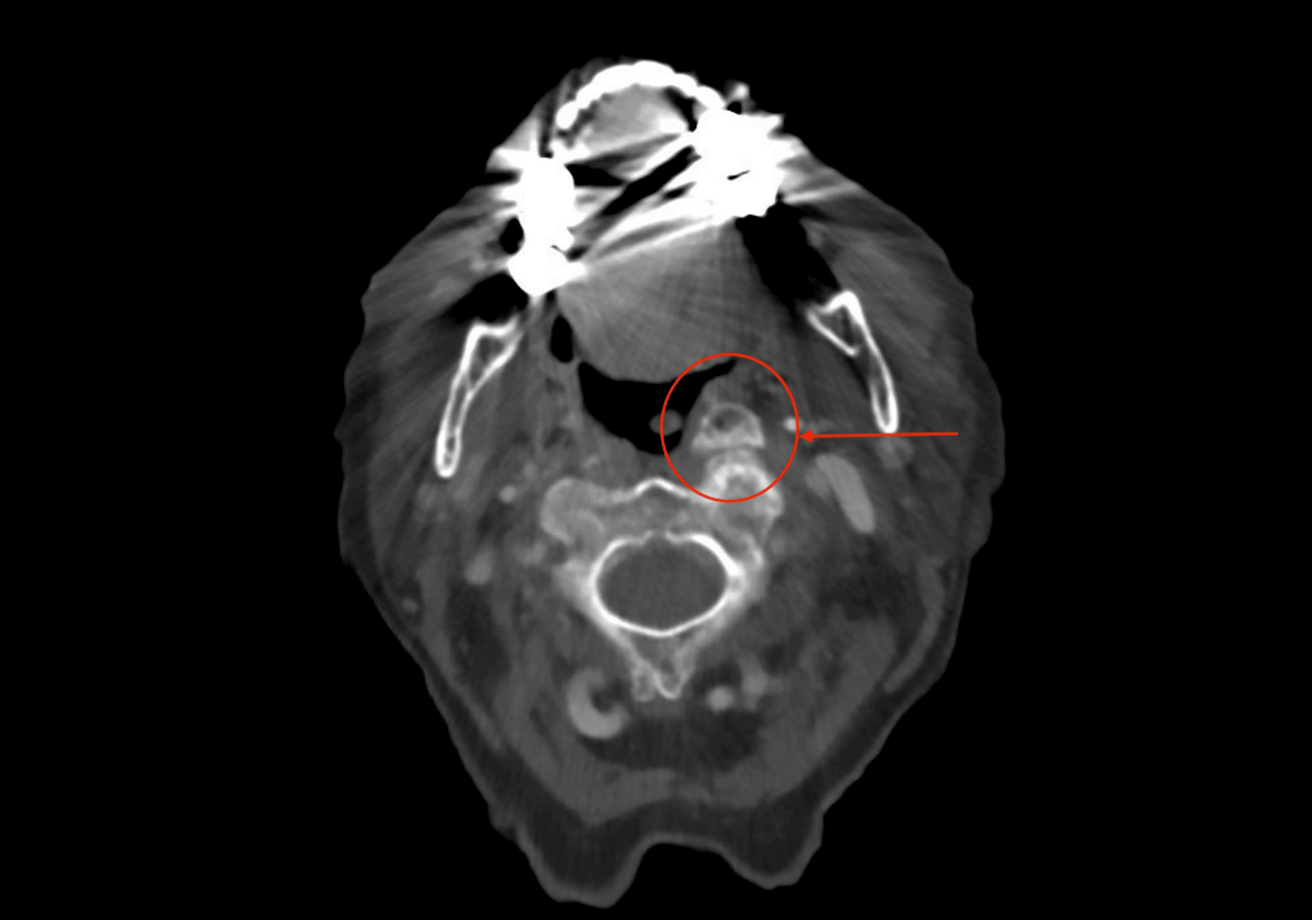
Transverse axial view demonstrating osteophyte with anteromedial displacement and mild narrowing of the oropharyngeal airway.

**Figure 3:**
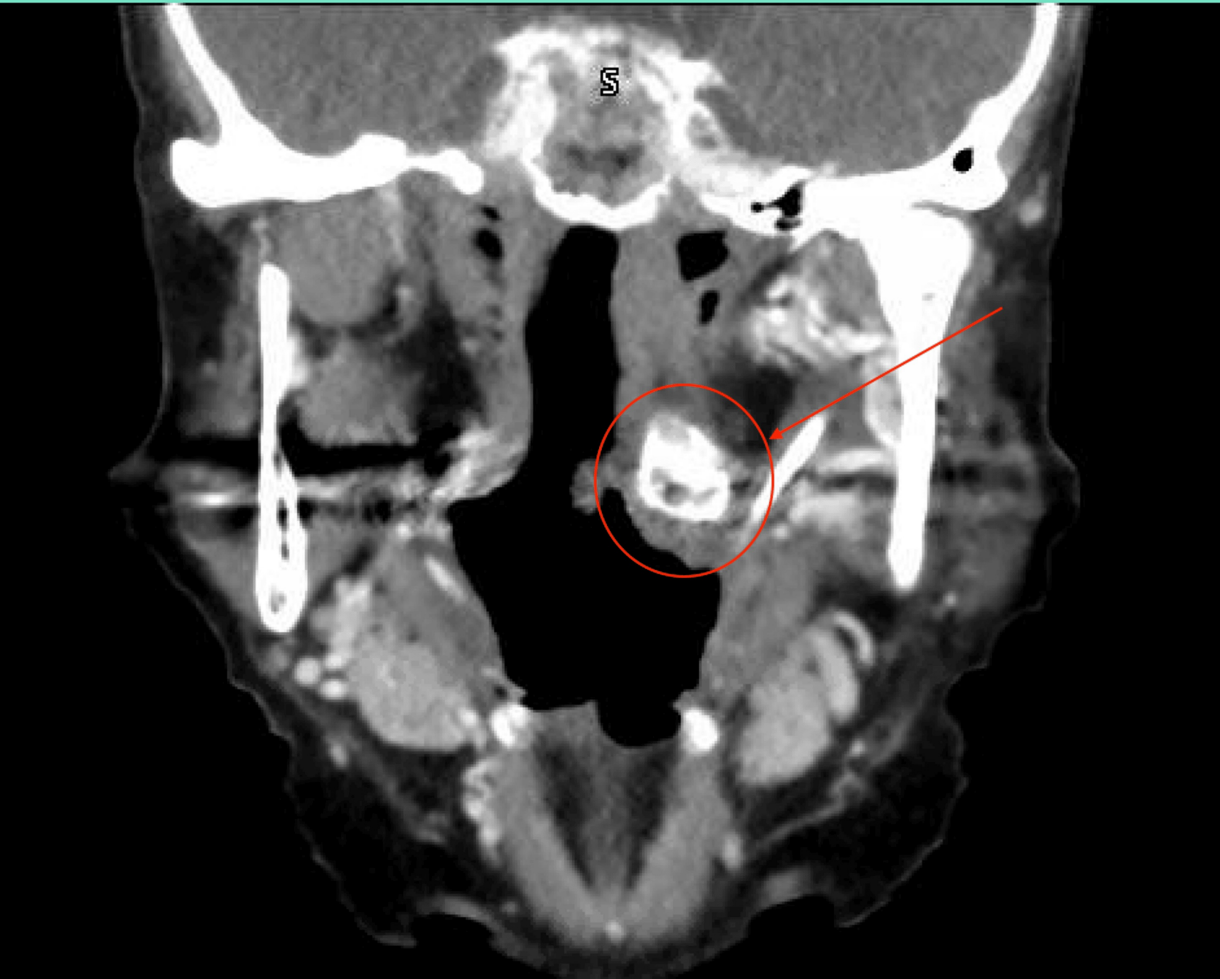
Coronal view demonstrating the cervical osteophyte with anteromedial displacement and mild narrowing of the oropharyngeal airway.

**Figure 4:**
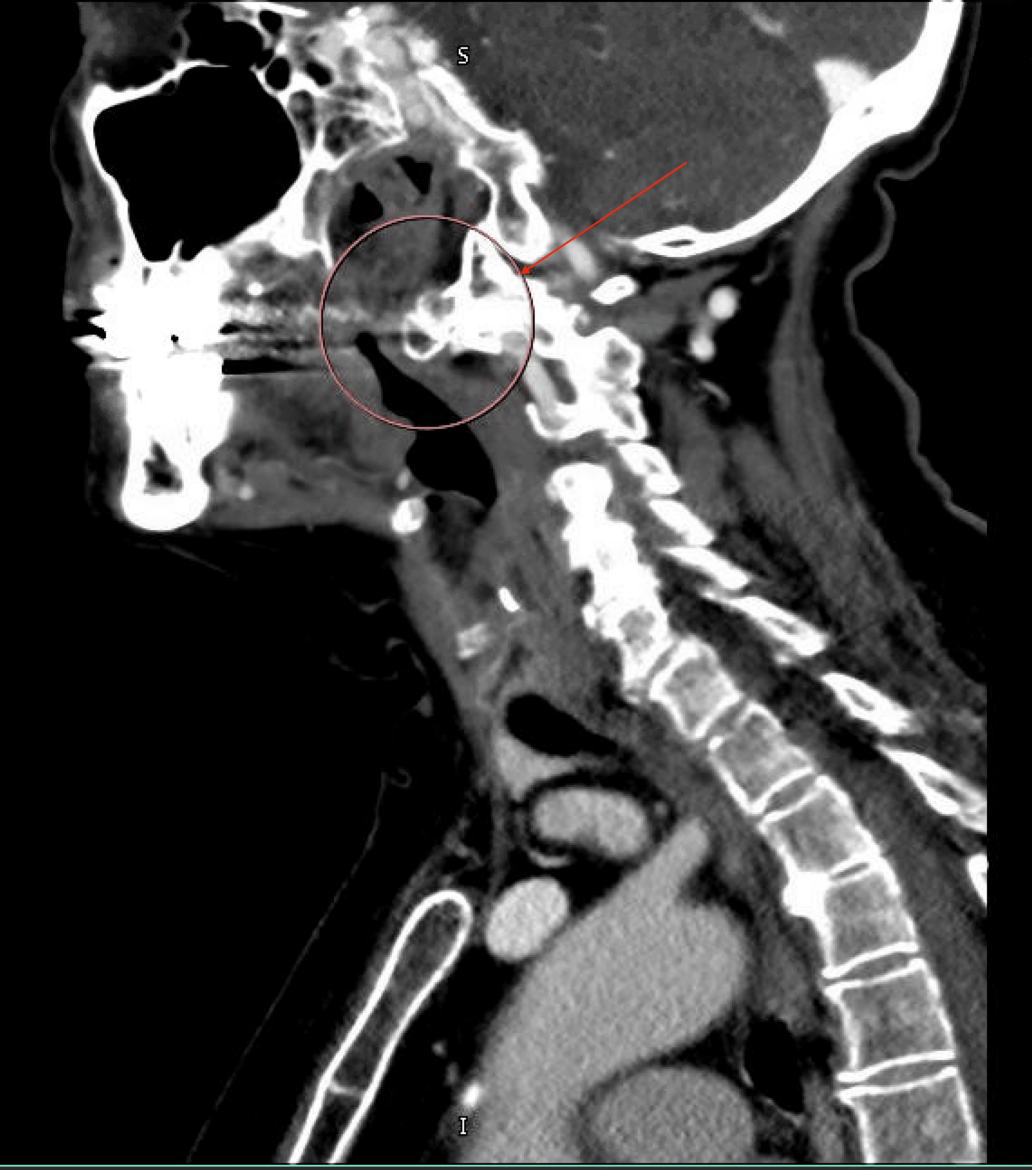
Sagittal view of the cervical osteophyte demonstrating anteromedial displacement and mild narrowing of the oropharyngeal airway.

A contrast-enhanced computed tomography (CT) scan of the neck revealed a bulky anterior osteophyte at the C1-C2 level on the left, which can be seen in Figures 2-4. This osteophyte exerted mass effect on the left palatine tonsil, causing anteromedial displacement and mild narrowing of the oropharyngeal airway. No enhancing mucosal lesions or suspicious lymphadenopathy were identified.

In the absence of radiographic or endoscopic evidence of malignancy, the tonsillar fullness was attributed to extrinsic compression from the cervical osteophyte.

## Discussion

Tonsillar asymmetry with associated firmness is a classic finding that raises suspicion for oropharyngeal malignancy, particularly squamous cell carcinoma or lymphoma. Evaluation typically includes a thorough physical examination, endoscopic assessment, and imaging when indicated.

Cervical osteophytes are a common manifestation of degenerative spinal disease, particularly in elderly patients. Approximately 95% of men and 70% of women older than 70 years demonstrate evidence of cervical spondylosis [6]. Prior literature describing symptomatic anterior cervical osteophytes has largely focused on presentations involving dysphagia, globus sensation, dysphonia, or airway compromise due to posterior pharyngeal wall or hypopharyngeal compression [7]. For example, Lin et al. described a case of hypertrophic anterior cervical osteophytes presenting as a hypopharyngeal mass with progressive dysphagia and airway compromise requiring surgical intervention [8]. In such cases, the mass effect is typically directed posteriorly, resulting in symptoms related to swallowing or airway obstruction rather than focal tonsillar findings.

In contrast, the present case demonstrates a unique presentation in which an osteophyte at the C1-C2 level produced localized anteromedial displacement of the palatine tonsil, mimicking a primary tonsillar mass on physical examination. Unlike previously reported cases, this patient did not exhibit significant dysphagia or airway compromise, and the primary clinical concern was malignancy due to tonsillar asymmetry and firmness. This highlights an atypical manifestation of cervical osteophytes and expands the spectrum of potential otolaryngologic presentations.

This case highlights the importance of imaging in patients with atypical presentations or discordant clinical findings. Reliance solely on physical examination may lead to unnecessary invasive procedures, such as biopsy or tonsillectomy. However, tissue diagnosis remains indicated in cases with persistent clinical concern, including progressive enlargement, mucosal abnormalities, associated cervical lymphadenopathy, or high-risk patient factors, even in the setting of non-diagnostic imaging. In appropriately selected patients, an imaging-first approach may help identify extrinsic causes of tonsillar asymmetry and reduce unnecessary surgical intervention, thereby minimizing procedural risk, anesthesia exposure, and associated healthcare costs. Clinical decision-making should therefore integrate examination findings, patient risk factors, and imaging results to guide the need for operative intervention.

## Conclusions

Cervical osteophytes should be considered in the differential diagnosis of tonsillar asymmetry, particularly in elderly patients. This case underscores the importance of cross-sectional imaging in distinguishing benign extrinsic compression from true mucosal pathology and avoiding unnecessary surgical intervention.
